# Brain metastases in patients with salivary duct carcinoma: A retrospective study

**DOI:** 10.1002/cam4.7037

**Published:** 2024-03-13

**Authors:** Chihiro Fushimi, Hideaki Takahashi, Daisuke Kawakita, Satoshi Kano, Kiyoaki Tsukahara, Hiroyuki Ozawa, Kenji Okami, Akihiro Sakai, Keisuke Yamazaki, Takuro Okada, Toyoyuki Hanazawa, Yuichiro Sato, Yorihisa Imanishi, Akira Shimizu, Takashi Matsuki, Toshitaka Nagao, Yuichiro Tada

**Affiliations:** ^1^ Head and Neck Oncology and Surgery International University of Health and Welfare, Mita Hospital Tokyo Japan; ^2^ Department of Head and Neck Surgery National Cancer Center Hospital Tokyo Japan; ^3^ Department of Otorhinolaryngology, Head and Neck Surgery Yokohama City University, School of Medicine Yokohama Japan; ^4^ Department of Otorhinolaryngology, Head and Neck Surgery Nagoya City University Graduate School of Medical Sciences Nagoya Japan; ^5^ Department of Otolaryngology Head and Neck Surgery, Faculty of Medicine and Graduate School of Medicine Hokkaido University Sapporo Japan; ^6^ Department of Otorhinolaryngology, Head and Neck Surgery Tokyo Medical University Tokyo Japan; ^7^ Department of Otorhinolaryngology Head and Neck Surgery Keio University School of Medicine Tokyo Japan; ^8^ Department of Otolaryngology Head and Neck Surgery Tokai University School of Medicine Isehara Japan; ^9^ Department of Head and Neck Surgery Niigata Cancer Center Hospital Niigata Japan; ^10^ Department of Otorhinolaryngology, Head and Neck Surgery Tokyo Medical University Hachioji Medical Center Tokyo Japan; ^11^ Department of Otolaryngology, Head and Neck Surgery Chiba University Graduate School of Medicine Chiba Japan; ^12^ Otorhinolaryngology, Head and Neck Surgery International University of Health and Welfare, Narita Hospital Chiba Japan; ^13^ Department of Otorhinolaryngology, Head and Neck Surgery Kitasato University School of Medicine Kanagawa Japan; ^14^ Department of Anatomic Pathology Tokyo Medical University Tokyo Japan

**Keywords:** androgen receptor, brain metastases, distant metastases, human epidermal growth factor receptor 2, salivary duct carcinoma

## Abstract

**Background:**

Salivary duct carcinoma (SDC) is a high‐grade adenocarcinoma with a 5‐year survival rate of 40%. Although drug therapy has improved patients' prognosis, the impact of brain metastasis (BM) remains poorly understood. We aimed to retrospectively examine the incidence of BM in patients with SDC (*n* = 464) and develop a tool to estimate their prognoses.

**Methods:**

We retrospectively examined 464 patients with SDC enrolled in a multicenter study. We investigated the incidence of BM, overall survival (OS) rates, and factors affecting prognosis in patients with BM. We also developed an SDC‐graded prognostic assessment (GPA) score for disease prognostication.

**Results:**

Sixty‐five (14%) patients had BM. The median OS (mOS) was 13.1 months. On univariate and multivariate analyses, factors such as Eastern Cooperative Oncology Group Performance Status >1, human epidermal growth factor receptor 2‐negative status, and locoregional uncontrolled disease were associated with poor OS. SDC‐GPA scores according to the prognostic factors were 0, 1, 2, and 3 points, and mOS estimates were 50.5, 16.1, 3.9, and 1.2 months, respectively (*p* < 0.001).

**Conclusion:**

The SDC‐GPA score emerged as a useful prognostication tool for patients with BM.

## 1 INTRODUCTION

Salivary duct carcinoma (SDC) is a rare type of cancer that is pathologically defined as a high‐grade adenocarcinoma that resembles mammary duct carcinoma.^1^, ^2^ The standard treatment for SDC is surgical resection and postoperative irradiation.^3^, ^4^, ^5^, ^6^ However, 40% of SDCs develop distant metastasis after the curative treatment, most commonly to the lung, bone, and liver,^7^, ^8^ resulting in a 5‐year survival rate of approximately 40%.^7^, ^8^, ^9^ One‐third of SDCs are positive for human epidermal growth factor receptor 2 (HER2), and almost all SDCs are positive for androgen receptor (AR), both of which are rarely found in other types of salivary gland cancer.^10^, ^11^, ^12^ Therefore, HER2‐ and AR‐targeted therapies are treatment options for these presentations.^13^, ^14^, ^15^, ^16^, ^17^

Brain metastasis (BM) is a relatively rare distant metastatic site in SDC; the incidence of BM ranges from 2% to 8% in patients with metastatic SDC.^8^, ^18^, ^19^ BM not only causes death but also results in personality changes, brain dysfunction, and impaired performance status (PS). Although survival in patients with BM has been estimated to be 3–6 months previously,^20^, ^21^ recent improvements in diagnostic imaging, radiation, and drug therapies have improved survival and quality of life.^22^, ^23^, ^24^, ^25^, ^26^, ^27^ However, the prognosis of patients with BM of SDC remains unclear since only a few case series have been reported.^28^, ^29^

In patients with BM originating from other types of cancers, such as lung and breast, the diagnosis‐specific graded prognostic assessment classification (DS‐GPA) uses factors associated with each type of cancer to aid the prognostication of BM.^30^ The DS‐GPA tool is useful for clinical decision‐making and stratification in clinical trials. However, the biological characteristics of BM in SDC remain unclear.

Therefore, we aimed to retrospectively examine the prognostic factors of BM in patients with SDC using an integrated database of SDC from seven institutions in Japan (*n* = 464), as well as treatment types, incidence, and outcomes. This is the first study to investigate the prognostic factors of BM in patients with SDC based on data from a multicenter study in Japan.

## 2 METHODS

### 2.1 Study design

This was a multicenter, retrospective study approved by the institutional ethics review board of each facility. Written informed consent for the publication of this study was obtained from all patients. A public opt‐out system for the residual use of patient data was used. All protocols were conducted in accordance with the Declaration of Helsinki.

### 2.2 Patients

This study included 464 patients diagnosed with SDC and treated at seven institutions between 1992 and 2020. Diagnostic accuracy was verified by an expert pathologist (TN) in accordance with the modern pathological criteria for SDC.^31^, ^38^, ^39^ Follow‐ups were conducted at each institution, and the patients' prognoses information was obtained from the medical records. Patients in this study have been included in previous studies.^7^, ^14^, ^17^, ^32^

### 2.3 Endpoints

The primary endpoint included factors associated with the prognosis of patients with BM. Secondary endpoints were BM incidence and overall survival (OS). A scoring system was developed for prognostication.

### 2.4 Statistical analysis

The incidence of BM was calculated as the percentage of all patients with SDC who had BM with or without other metastases. The time to BM was calculated from the start of the first treatment to the day when the evidence of BM was first observed through imaging. One patient with BM at initial diagnosis was excluded from these calculations.

Overall survival was defined as the day of BM diagnosis until the day of death from any cause. OS was estimated using the Kaplan–Meier method. The relationships between age, sex, PS, stage, symptoms at BM diagnosis, number of metastatic lesions, primary site, HER2 status, and AR status were assessed using univariate and multivariate Cox regression models. HER2 and AR statuses were determined according to a previously reported method.^11^, ^12^ The SDC‐GPA score ranged from 0 to +3, with each prognostic factor considered significant using multivariate analysis graded 0 or +1. The association between the total score (summary of each prognostic factor grade) and OS was examined. All statistical analyses were performed using EZR (Saitama Medical Center, Jichi Medical University, Saitama, Japan).

## 3 RESULTS

### 3.1 Rate of BMs

**FIGURE 1:**
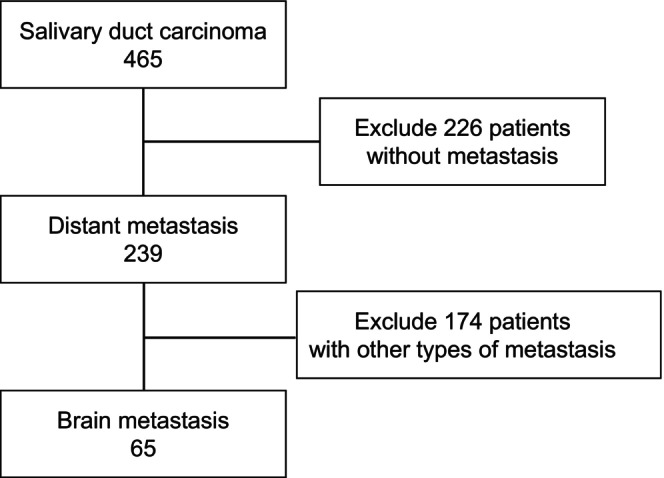
Flowchart.

The median follow‐up period of 464 cases was 34.4 months (range, 0.5–227.6 months). Among these patients, 239 (51.4%) had distant metastases, including 65 (14.0%) with BM (Figure 1). The median time to develop BM from the initial treatment was 20.0 months (range, 1.7–83.2 months).

### 3.2 Clinical characteristics of patients with BMs

Among the 65 patients with BM, the median follow‐up period after the development of BM was 11.2 months (range, 0.2–66.5 months) (Table 1). The median age was 61 years, and 55 (85%) patients were male. Most patients had an Eastern Cooperative Oncology Group (ECOG) PS of 0 or 1 (77%). The most common primary tumor site was the parotid gland (65%). Stage III/IV disease was observed in 80% of the patients. Only 5% of patients had BM alone; moreover, 95 patients had other DM, especially in the lung and liver. Additionally, locoregional control was achieved in 70% of the patients during BM detection. The rates of HER2‐ and AR‐positivity were 67% and 95%, respectively. HER2 positivity comprised an IHC score of 3+ or 2+ (93%) and FISH positivity (7%). Overall, 83% and 38% of the patients had multiple and symptomatic BM, respectively.

**TABLE 1:** Patient characteristics (*n* = 65).

Median age, years (range)	61 [26–84]
Sex, *n* (%)	
Male	55 (85)
Female	10 (15)
ECOG performance status, *n* (%)	
0	30 (46)
1	20 (31)
2	7 (11)
3	8 (12)
Primary tumor site, *n* (%)	
Parotid grand	42 (65)
Submandibular grand	22 (34)
Minor salivary gland	1 (1)
Stage at diagnosis of cancer^a^, *n* (%)	
I/II	13 (20)
III/IV	52 (80)
Clinical symptoms, *n* (%)	
Yes	24 (38)
No	39 (62)
Other metastases, *n* (%)	
Yes	60 (95)
No	3 (5)
HER2 status^b^, *n* (%)	
Positive	43 (67)
Negative	21 (33)
Primary site control, *n* (%)	
Yes	44 (70)
No	19 (30)
Brain metastases, *n* (%)	
Single	11 (17)
Multiple	54 (83)

Abbreviations: ECOG, Eastern Cooperative Oncology Group; HER2, human epidermal growth factor receptor 2.

^a^ According to the Union for International Cancer Control and TNM classification and staging system (2017, 8th edition).

^b^ According to the American Society of Clinical Oncology/College of American Pathologists guidelines for HER2 testing.

### 3.3 Treatment

Among the patients with BM (*n* = 65), 35, 10, 1, 1, and 15 were treated with stereotactic irradiation, whole brain irradiation, surgery plus irradiation, cytotoxic chemotherapy alone, and best supportive care, respectively. Treatment modalities were unknown in three cases. Overall, radiotherapy was administered to 45 (74.2%) patients. Systemic therapy was used in 42 (64.6%) patients after the initial treatment for BM, including trastuzumab and docetaxel (Tmab/DTX), combined androgen blockade (CAB), cytotoxic chemotherapy, and unknown agents in 23 (54.8%), 7 (16.7%), 10 (23.8%), and 2 (4.8%) patients, respectively. AR‐positive/HER2‐positive SDC were frequently treated with either CAB or HER2‐targeted therapy, with the trend leaning toward HER2‐targeted therapy due to its higher efficacy.

### 3.4 OS in patients with BMs

**FIGURE 2:**
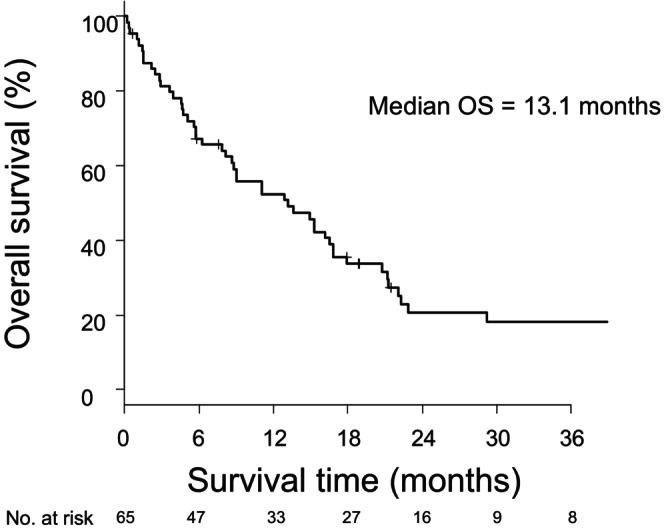
Kaplan–Meier curve for survival of patients with brain metastasis.

The median OS (mOS) was 13.1 (95% confidence interval [CI], 8.1–16.8) months (Figure 2). The 1‐ and 3‐year OS rates were 52.4% (95% CI, 39.4%–63.9%) and 18.3% (95% CI, 9.1%–29.9%), respectively.

### 3.5 Prognostic factors

Univariate analysis showed that factors, including ECOG PS ≥1, symptomatic BM, HER2‐negative status, and uncontrolled locoregional region, were associated with poor OS. Multivariate analysis showed that ECOG PS ≥1, HER2‐negative status, and locoregionally uncontrolled disease were associated with poor OS (Table 2).

**TABLE 2:** Factors associated with overall survival in univariate and multivariate analysis.

Characteristics	*n*	Univariate			Multivariate		
		HR	95% CI	*p* Value	HR	95% CI	*p* Value
Age							
<60 years	29	1	Ref	0.36	1	Ref	0.52
≥60 years	36	1.42	0.67–2.99		1.23	0.65–2.30	
Sex							
Female	10	1	Ref	0.76	1	Ref	0.65
Male	55	0.86	0.33–2.24		1.21	0.52–2.81	
ECOG performance status							
0	30	1	Ref	**0.0036**	1	Ref	**0.00017**
1–3	35	3.50	1.51–8.12		3.74	1.88–7.44	
Primary tumor site							
Others	23	1	Ref	0.97			
Parotid grand	42	1.01	0.49–2.11				
Stage at diagnosis							
III/IV	59	1	Ref	0.11			
I/II	6	2.68	0.79–9.08				
Clinical symptoms							
No	39	1	Ref	**0.043**	1	Ref	0.14
Yes	24	2.09	1.02–4.28		1.59	0.86–2.95	
Other metastases							
No	3	1	Ref	0.81			
Yes	60	1.20	0.26–5.45				
HER2 status							
Positive	43	1	Ref	**0.007**	1	Ref	**0.012**
Negative	21	2.72	1.32–5.64		2.29	1.20–4.36	
Primary site control							
Yes	44	1	Ref	**0.000046**	1	Ref	**0.000018**
No	19	5.55	2.44–12.67		5.20	2.45–11.05	
Brain metastases							
Single	11	1	Ref	0.12			
Multiple	54	2.59	0.78–8.65				

Abbreviations: ECOG, Eastern Cooperative Oncology Group; HER2, human epidermal growth factor receptor 2; HR, hazard ratio; OS, overall survival. Boldfaced *p* values indicate.

### 3.6 SDC‐GPA score

**FIGURE 3:**
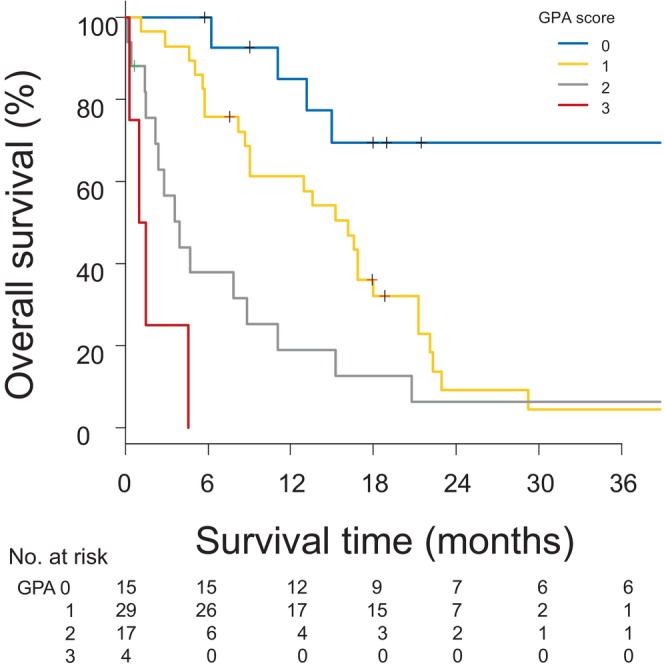
Kaplan–Meier curve for survival of patients with brain metastasis by SDC‐GPA score SDC, salivary duct carcinoma; GPA, graded prognostic assessment.

ECOG PS ≥1, HER2‐negative status, and locoregional uncontrolled disease were each scored as 1 to obtain an SDC‐PGA score, where a score of 3 correlates with the worst prognosis (Table 3). In this study, 15, 29, 17, and 4 patients were scored as 0, 1, 2, and 3 points, respectively, and the corresponding mOS were 50.5 months (95% CI, 13.2 months–not reached), 16.1 months (95% CI, 8.7–17.9 months, HR, 3.65; 95% CI: 1.53–8.73; *p* = 0.004), 3.9 months (95% CI, 1.5–8.8 months, HR, 7.09; 95% CI: 2.88–17.46; *p* < 0.001), and 1.2 months (95% CI, 0.3 months–not reached, HR, 42.23; 95% CI: 10.48–170.20; *p* < 0.001), respectively (Table 4, Figure 3).

**TABLE 3:** The factors of graded prognostic assessment (GPA) for salivary duct carcinoma.

Factor	GPA scoring	
	0	+1
HER2	Positive	Negative
ECOG PS	0	1–3
Primary control	Yes	No

Abbreviations: ECOG, Eastern Cooperative Oncology Group; HER2, human epidermal growth factor receptor 2.

**TABLE 4:** Univariate analysis of graded prognostic assessment for salivary duct carcinoma and overall survival.

GPA	*N* (%)	Median OS (months) (95% CI)	HR (95% CI)	*p*
0	15 (23)	50.5 (13.2–NR)	1.00	–
1	29 (45)	16.1 (8.7–17.9)	3.65 (1.53–8.73)	0.0036
2	17 (26)	3.9 (1.5–8.8)	7.09 (2.88–17.46)	<0.001
3	4 (6)	1.2 (0.3–NR)	42.23 (10.48–170.20)	<0.001

Abbreviations: GPA, graded prognostic assessment; OS, overall survival; HR, hazard ratio, NR, not reached.

## 4 DISCUSSION

In this study of 464 individuals with SDC, BM occurred in 14.0% of all patients, and the mOS after BM was 13.1 months. Furthermore, this study showed that ECOG PS ≥1, HER2‐negative status, and locoregional uncontrolled disease were associated with poor survival. A four‐level SDC‐GPA score was constructed based on these factors, showing good predictive value.

The percentage of patients with parotid gland carcinoma with BM at the first visit was 1.6%–6.0%, according to the National Cancer Database registry (2010–2015).^19^, ^33^ Meanwhile, among 154 patients with SDC with distant metastasis, 9 (7%) had BM.^18^ In another previous study, BM occurred in 15 of the 177 (8%) patients with SDC.^8^ However, in this study, the incidence of BM among patients with SDC was 14.0%, which is higher than that previously reported. This study included patients who received Tmab/DTX. Our previous prospective studies showed a response rate and mOS of 70.2% and 39.7 months, respectively, in patients with HER2‐positive SDC treated with Tmab/DTX.^14^ Additionally, it has been reported that Tmab/DTX prolonged OS in patients with recurrent metastatic SDC.^37^ Overall, the number of patients with BM may have increased because Tmab/DTX has prolonged OS, while Tmab failed to control BM due to its inability to cross the blood–brain barrier. In our institutions, postoperative MRIs are not always performed on patients with SDC. In this study, only 14 cases were found to have brain metastases based on routine MRI. Many other cases were either symptomatic or discovered incidentally on follow‐up CT. However, it has recently been shown that brain metastases are more common when distant metastases involving the lungs have occurred. Therefore, MRI may have been performed more frequently in such cases at our institution.

To the best of our knowledge, this is the first study to investigate the prognostic factors of BM in patients with SDC. A study of patients with parotid gland carcinoma with BM at the initial presentation reported a mOS of 8.3 months (95% CI, 5.6–11.0).^19^ Although direct comparisons are difficult, the mOS after BM in this study was 13.1 months, showing some improvement compared to that previously reported. This finding could be explained by the fact that patients with BM are likely to present with more aggressive or advanced disease than those without BM. In this study, only one patient had BM during the initial diagnosis, which may have affected the results. These findings may also be explained by the differences in treatment rates and methods. Messing et al. reported on 62.7% and 50.2% of patients treated with radiation and systemic therapies, respectively, and the corresponding rates in our study were 74.2% and 61.5%, respectively. In our study, more patients received treatment, including Tmab/DTX (54.8%) and CAB (16.7%), than those in the previous study. Tmab/DTX and CAB therapies may have reduced the number of deaths due to factors other than BM, increasing the mOS estimated in patients with BM.

In a previous study, Sperduto et al.^30^ proposed that GPA scores based on age, Karnofsky Performance Status, number of metastases, and extracranial metastases may help prognosticate outcomes in patients with BM from lung cancer, malignant melanoma, breast cancer, renal cell cancer, and gastrointestinal cancer. In this study, factors associated with poor prognosis included ECOG PS >1, HER2‐negative status, and locoregional uncontrolled disease.

ECOG PS is a prognostic factor for many types of cancer. In this study, 95% of the patients with BM had distant metastases outside of the brain. The poor prognosis of patients with low ECOG PS may be due to other metastases that remain inadequately controlled because of systemic therapy deficiencies, even in cases where BM is controlled by radiotherapy.

The prognostic value of HER2‐negativity in this study was similar to that in patients with breast cancer with BM.^34^, ^35^ The American Society of Clinical Oncology guidelines for breast cancer recommend surgery or radiation therapy followed by appropriate anti‐HER2 therapy for BM in patients with HER2‐positive breast cancer.^36^ In this study, among the 43 patients with HER2‐positive BM, 23 were treated with Tmab/DTX, suggesting treatment overlap between breast cancer and SDC.

The impact of locoregional uncontrolled disease was comparable to that of other carcinomas in a study by Sperduto et al.^30^ Locoregional uncontrolled disease during BM diagnosis suggests previous treatment failure. Even if the treatment for BM is successful, the prognosis of patients remains poor due to factors other than BM. Therefore, improved therapies are required to prolong survival in this patient group.

In this study, the number of BMs and other DMs was not a predictive factor for OS, as observed in other cancers. This could be attributed to the fact that SRT was effective even with multiple BMs; additionally, HER2/AR therapy may have been relatively more successful for distant metastases.

In this study, we developed the SDC‐GPA score for prognostication in patients with BM based on ECOG PS, HER2 status, and locoregional control. For all patients with cancer with BM, the prognosis should be assessed, and treatments should be decided during diagnosis, as in cases of breast and lung cancer. If the prognosis is favorable, aggressive treatment is recommended by the guidelines for brain tumor treatment. However, if the prognosis is poor, including an SDC‐GPA score of 2 or 3 points, invasive treatments such as surgery should be avoided. However, further studies on SDC‐GPA scores and treatment are needed.

This study has some limitations. First, this was a retrospective study, and some bias was inevitable in the assessment of treatment type, frequency, and efficacy, specifically in cases that reported curative effects. Second, the SDC‐GPA score was derived from a sample that included patients eligible for anti‐HER2 treatment for recurrent SDC; however, this treatment may not be easily accessible in other contexts.^34^ Third, any changes to treatment protocols may affect factors accounted for in the score. Finally, although this study is the largest reported to date on patients with SDC (*n* = 65), the sample size remains small compared to those observed in other cancer types. Therefore, analysis of additional cases is required to validate these findings.

## 5 CONCLUSIONS

In this study, we examined 65 patients who had SDC with BM. The rate of BM and mOS were 14% and 13.1 months, respectively. Multivariate analysis showed that ECOG PS >1, HER2‐negative status, and locoregional uncontrolled disease were associated with poor OS. Furthermore, the SDC‐GPA score showed predictive value in the prognostication of BM in SDC. These findings may inform future treatment for BM and help improve patient quality of life.

## AUTHOR CONTRIBUTIONS

**Chihiro Fushimi:** Conceptualization (equal); funding acquisition (equal); methodology (equal); supervision (equal); writing – original draft (equal); writing – review and editing (equal). **Hideaki Takahashi:** Conceptualization (equal); data curation (equal). **Daisuke Kawakita:** Software (equal); supervision (equal); writing – review and editing (equal). **Satoshi Kano:** Data curation (equal); writing – review and editing (equal). **Kiyoaki Tsukahara:** Data curation (equal); methodology (equal); writing – review and editing (equal). **Hiroyuki Ozawa:** Data curation (equal); writing – review and editing (equal). **Kenji Okami:** Data curation (equal); validation (equal); writing – review and editing (equal). **Akihiro Sakai:** Data curation (equal); resources (equal); writing – review and editing (equal). **Keisuke Yamazaki:** Data curation (equal); writing – review and editing (equal). **Takuro Okada:** Data curation (equal); writing – review and editing (equal). **Toyoyuki Hanazawa:** Data curation (equal); formal analysis (equal); writing – review and editing (equal). **Yuichiro Sato:** Data curation (equal); writing – review and editing (equal). **Yorihisa Imanishi:** Data curation (equal); writing – review and editing (equal). **Akira Shimizu:** Data curation (equal); writing – review and editing (equal). **Takashi Matsuki:** Data curation (equal); writing – review and editing (equal). **Toshitaka Nagao:** Conceptualization (equal); data curation (equal); resources (equal); writing – original draft (equal); writing – review and editing (equal). **Yuichiro Tada:** Conceptualization (equal); supervision (equal); writing – original draft (equal); writing – review and editing (equal).

## FUNDING INFORMATION

This work was supported by JSPS Grants‐in‐Aid for Scientific Research (C) to Dr. Yuichiro Tada (No. 21K09616), Dr. Chihiro Fushimi (No. 21K16835), Dr. Takashi Matsuki (No. 20K16835), and Dr. Toshitaka Nagao (No. 20K07417).

## CONFLICT OF INTEREST STATEMENT

The authors declare no conflicts of interest.

## ETHICS STATEMENT

This study was approved by the ethics committee of the International University of Health and Welfare Mita Hospital (H23‐12).

## CONSENT STATEMENT

Written informed consent for the publication of the present study was obtained from all patients. A public opt‐out system for the residual use of patient data was used.

## Supporting information


Appendix S1

## Data Availability

The data that support the findings of this study are available from the corresponding author upon reasonable request.
