# Supplementary material for: Brain metastases in patients with salivary duct carcinoma: A retrospective study
Source: Cancer Med. 2024 Mar 13;13(5):e7037. doi: 10.1002/cam4.7037 (PMC10935879; doi:10.1002/cam4.7037)
Supplement: Supplementary file 1 — Appendix S1 [file CAM4-13-e7037-s001.docx]

supplementary data

Table S1: The time from primary treatment initiation to the diagnosis of BM and OS according to the cT factors.

| Factor | Group | n | Median time to BM (range) | p-value |
| --- | --- | --- | --- | --- |
| cT | 1 | 5 | 544 (286–1141) | 0.448 |
|  | 2 | 22 | 936 (52–2498) |  |
|  | 3 | 7 | 523 (193–1168) |  |
|  | 4a | 26 | 541 (105–1300) |  |
|  | 4b | 4 | 527 (204–1010) |  |
| T1-3 vs. T4a, 4b | T1-3 | 34 | 742 (52–2498) | 0.337 |
|  | T4a, T4b | 30 | 554 (105–1300) |  |

BM, brain metastasis

Table S2: Overall Survival according to cT factors.

| Factor | Group | n | Median OS (95% CT) | p-value |
| --- | --- | --- | --- | --- |
| cT | 1 | 5 | 2.9 (1.1–NA) | 0.898 |
|  | 2 | 22 | 8.5 (4.7–16.8) |  |
|  | 3 | 7 | 16.1 (5.6–NA) |  |
|  | 4a | 26 | 15.3 (8.7–22.1) |  |
|  | 4b | 4 | 13.9 (4.6–NA) |  |
| cT1-3 vs. T4a, 4b | T1-3 | 34 | 10.0 (5.6–16.8) | 0.565 |
|  | T4a, T4b | 30 | 15.2 (8.67–22.1) |  |

OS, overall survival; CT, computed tomography

Legends:

Fig. S1: Kaplan-Meier Curve of overall survival based on cT stages.

Fig. S2: Kaplan-Meier Curve of overall survival based on cT stage 1-3 vs. cT stage 4.


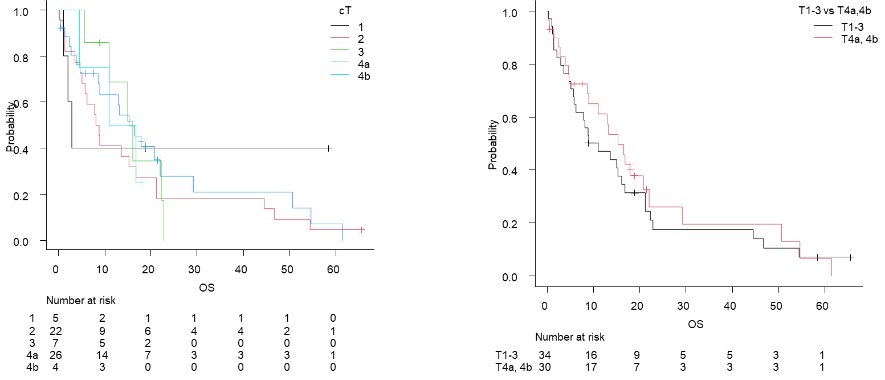


Fig. S1 Fig. S2.
